# Internet gaming disorder, psychological distress, and insomnia in adolescent students and their siblings: An actor-partner interdependence model approach

**DOI:** 10.1016/j.abrep.2020.100332

**Published:** 2020-12-29

**Authors:** Chung-Ying Lin, Mac N. Potenza, Anders Broström, Amir H. Pakpour

**Affiliations:** a Institute of Allied Health Sciences, National Cheng Kung University Hospital, College of Medicine, National Cheng Kung University, Tainan, Taiwan; b Departments of Psychiatry and Neuroscience and the Child Study Center, School of Medicine, Yale University, New Haven, CT 06511, USA; c Connecticut Council on Problem Gambling, Wethersfield, CT 06109, USA; d Department of Nursing, School of Health and Welfare, Jönköping University, Jönköping, Sweden; e Social Determinants of Health Research Center, Research Institute for Prevention of Non-Communicable Diseases, Qazvin University of Medical Sciences, Qazvin, Iran

**Keywords:** Adolescence, Anxiety, Depression, Online gaming, Sleep, Addictive behaviors

## Abstract

**Background:**

Associations between internet gaming disorder (IGD), psychological distress, and sleep have been reported. However, little is known whether such associations exist across siblings; that is, whether adolescents’ IGD symptomatology may impact their siblings’ psychological distress and sleep. This study aimed to examine whether siblings' IGD symptoms may relate to depressive, anxiety symptoms or sleep quality among each other.

**Methods:**

Using a cross-sectional design with two-stage cluster sampling, 320 dyads of adolescent students and their siblings participated in the study. Each dyad completed the Internet Gaming Disorder Scale-Short Form (IGDS-SF9), the Depression Anxiety Stress Scale-21 (DASS-21), and the Insomnia Severity Index (ISI). The actor–partner interdependence model (APIM) was applied to examine relationships between IGD, psychological well-being, and insomnia severity in the dyadic data.

**Results:**

Actor effects of IGDS-SF9 scores on depression, anxiety, stress, and insomnia severity were significant in both adolescents (e.g., adolescents’ IGDS-SF9 scores on their depression scores) and their siblings (e.g., IGDS-SF9 scores of adolescents’ siblings’ scores on their depression scores). Partner effects of IGDS-SF9 scores on depression, anxiety, stress, and insomnia severity were significant in both adolescents (e.g., adolescents’ IGDS-SF9 scores on their siblings’ depression scores) and their siblings (e.g., IGDS-SF9 scores of adolescents’ siblings on adolescents’ depression scores).

**Conclusions:**

The present study demonstrated that adolescent students and their siblings had mutual impacts of IGD on psychological health and sleep. Thus, healthcare providers may consider involving siblings when they design programs reducing IGD-related problems or improving psychological health and sleep for adolescents.

## 1 Introduction

Internet gaming disorder (IGD), a tentative psychiatric disorder defined by the *Diagnostic and Statistical Manual of Mental Disorders (DSM–5)* (American Psychiatric Association, 2013), has been studied with growing interest (Chen et al., 2020b, Leung et al., 2020, Pontes and Griffiths, 2015, Pontes et al., 2017, Wu et al., 2017). Associations between IGD and psychological distress have been documented. For example, IGD has been associated with depression, anxiety, obsessive–compulsive behavior, attention deficit/hyperactivity, poor self-esteem, loneliness, and social phobia (Andreassen et al., 2016, Cole and Hooley, 2013, Hyun et al., 2015, King and Delfabbro, 2016, Laconi et al., 2017, Wang et al., 2019).

Associations between IGD and sleep have also been documented. For example, IGD has been associated with poor sleep quality, daytime sleepiness, and insomnia (Alimoradi et al., 2019, Fossum et al., 2014, Wong et al., 2020). IGD may contribute to poor sleep for the following reasons. First, individuals with IGD may receive psychological stimulation (e.g., excited mood due to gaming) that reduces their sleepiness at bedtimes (Hale et al., 2018). Second, following reduced sleepiness, individuals with IGD may have delayed bedtimes and later waking times, which may subsequently lead to the rhythm desynchronization and insomnia (Touitou et al., 2016, Van den Bulck, 2004). Third, for those using smartphone to play games, they may be disturbed by light-emitting screens; that is, blue light from smartphones may suppress their sleep-promoting hormones (e.g., melatonin) (van der Lely et al., 2015). Moreover, empirical findings reveal the association between online gaming and shorter total rapid eye movement sleep with longer sleep latencies (Higuchi, Motohashi, Liu, & Maeda, 2005).

Although relationships between IGD, psychological distress, and sleep have been investigated, to the best of our knowledge, no studies have examined such topics on adolescents together with their siblings, especially those who share a bedroom. According to attachment theory, a child’s emotional development is highly associated with his or her parents (or caregivers) (Bowlby, 1969), and Ainsworth (1989) extended the attachment theory to emphasize the importance of siblings. Specifically, siblings may function as a secure base for an adolescent because siblings may provide more unconditional support, positive appraisal, and encouragement (Mota & Matos, 2015). Moreover, siblings often accompany each other with intense shared experiences and emotions (Dunn, 2000). Therefore, it is likely that siblings are important factors in associations between IGD, psychological distress, and sleep. For example, siblings may share experiences in gaming and even form a team in gaming; their bedtime and sleep quality may be associated when they share a bedroom. However, it is unclear whether an adolescents’ IGD may impact on their siblings’ psychological distress and sleep, and *vice versa*.

In order to address this literature gap, this study used an advanced statistical method that considered the nature of dyadic data (e.g., adolescents and their siblings in the present data) to investigate whether mutual impacts of IGD on psychological distress and sleep exist. Therefore, we proposed the following hypotheses: (1) adolescents’ levels of IGD associate with their siblings’ psychological distress and insomnia severity; (2) siblings’ levels of IGD associate with the adolescents’ psychological distress and insomnia severity; (3) adolescents’ levels of IGD associate with their own psychological distress and insomnia severity; and, (4) siblings’ levels of IGD associate with their own psychological distress and insomnia severity.

## 2 Methods

### 2.1 Participants and procedure

This cross-sectional study was conducted on 320 adolescent students and their siblings from October 2019 to February 2020. A two-stage cluster sampling was used to recruit adolescent students in Qazvin, Iran. In the first stage, ten schools were randomly selected from the list of all 56 Qazvin high schools. In the second stage, two classes were randomly selected from each school. All students from these classes were assessed for eligibility. The inclusion criteria were (1) being aged between 13 and 18 years, inclusive; (2) having the ability to understand written Persian; (3) possessing a smartphone because possessing a smartphone increase the likelihood of gaming with friends online (however, we have also assessed whether the participants played other games via personal computers or gaming consoles); (4) having access to the internet; (5) having a sibling aged between 13 and 24 years old; and (6) sharing a bedroom with the sibling. Apart from age, the siblings should fulfill the same inclusion criteria of the eligible participant. If the adolescent student had several siblings, we selected the sibling closest to the adolescent’s age. After completing the study, we provided a free healthy diet app with 3 months’ access to internet to both adolescents and their siblings. They were clearly informed that they could withdraw from the study anytime without any punishment.

All adolescents and their siblings (and their parents) gave informed consent to participate in the study. The study was approved by the Ethics Committee of Qazvin University of Medical Sciences (no. IR.QUMS.REC.1398.416) and the Organization for Education in Qazvin.

### 2.2 Instruments

Both participants and their 13- to 24-year-old siblings completed the following instruments.

#### 2.2.1 Internet Gaming Disorder Scale-Short Form (IGDS-SF9)

The IGDS-SF9 is a nine-item questionnaire that assesses severity of IGD (Pontes & Griffiths, 2015). The nine items are rated on a 5-point Likert scale (total scores range between 9 and 45) and correspond to the IGD criteria defined by the *Diagnostic and Statistical Manual of Mental Disorders, 5th edition* (DSM-5; American Psychiatric Association, 2013). A higher total score indicates greater severity. The psychometric properties of the IGDS-SF9 are sound (Chen et al., 2020b, Leung et al., 2020, Pontes et al., 2017), including the Persian version (Wu et al., 2017).

#### 2.2.2 Depression Anxiety Stress Scale-21 (DASS-21)

The DASS-21 is a 21-item questionnaire that assesses psychological distress in terms of depression, anxiety, and stress (Lovibond & Lovibond, 1995). The 21 items are distributed into three subscales (depression, anxiety, and stress) and rated on a 4-point Likert scale (subscale score ranges between 0 and 21). Higher subscale scores indicate more severe depression, anxiety, or stress. The psychometric properties of the DASS-21 are sound (Shaw et al., 2017, Wang et al., 2016), including the Persian version (Asghari, Saed, & Dibajnia, 2008).

#### 2.2.3 Insomnia Severity Index (ISI)

The ISI is a seven-item questionnaire that assesses insomnia severity (Chiu, Chang, Hsieh, & Tsai, 2016). The seven items are rated on a 5-point Likert scale (total score ranges between 0 and 28) and emphasizes worry about sleep and evidence of sleep problems (Chung, Kan, & Yeung, 2011). A higher score indicates more severe insomnia. The psychometric properties of the ISI are sound (Morin et al., 2011, Savard et al., 2005), including the Persian version (Lin et al., 2020, Yazdi et al., 2012).

### 2.3 Data analysis

Descriptive data were analyzed in forms of percentages, means, and standard deviations. The adolescent students’ and their siblings’ differences in sociodemographic characteristics were compared using chi-square tests for categorical variables and t-tests for continuous variables. Correlations between variables were calculated using Pearson correlation coefficients (r’s). Moreover, an actor–partner interdependence model (APIM), which accounts for interdependence within close relationships in a statistical model, was applied to investigate our hypotheses.

Specifically, the APIM (Kenny, Kashy, & Cook, 2006) was used to investigate if IGD in adolescent students and their siblings were associated with their own (i.e., actor) and their siblings’ (i.e., partner) psychological distress and insomnia. The APIM is a model of dyadic relationships that tests a model of interdependence within close relationships. The APIM includes two major effects: intrapersonal effects (actor effects) and interpersonal effects (partner effects). Therefore, the APIM is able to measure effects between two variables within an individual (i.e., actor effect) and simultaneously accounting for interpersonal relationship regarding his/her partner’s outcome (i.e., partner effect; Cook & Kenny, 2005). Four APIMs incorporated with structural equation models (SEMs) were conducted, of which IGD was the independent variable across the four models; insomnia, depression, anxiety, and stress were dependent variables for each model. Moreover, the collinearity between the independent variables (i.e., the two IGD variables) was considered using their correlations estimated in the SEM. The APIM SEM was estimated using a maximum likelihood (ML) method. Additionally, the APIM SEM examined whether the dyads are distinguishable (i.e., distinguished by their role as an adolescent student or as a sibling). In all APIM models, sociodemographic covariates (i.e., gender and age of adolescent students and their siblings) were controlled.

All analyses were performed by the online app (APIM_SEM) developed by Stas, Kenny, Mayer, and Loeys (2018) using the R-package lavaan (Rosseel, 2012), an R-package for SEM.

## 3 Results

### 3.1 Demographics and correlation

Among the 640 dyads of adolescent students and their siblings, the siblings were significantly older than the adolescent students. The sibling group contained more females relative to the adolescent student group (Table 1). The adolescent students as compared with their siblings had higher levels of psychological distress and lower levels of severity of insomnia. No between-group differences were found for IGDS-SF9 scores and time spent gaming (Table 1). Table 2 presents the correlation matrix between variables.

**Table 1:** Participant characteristics (N = 640).

	Mean ± SD or n (%)		t-value or χ^2^	P value
	Adolescent student (n = 320)	Sibling (n = 320)		
Age (Years)	15.52 ± 1.98	16.98 ± 2.91	8.295	<0.001
Gender (Male)	169 (52.8)	255 (79.7)	51.684	<0.001
Score on Internet Gaming Disorder Scale-Short Form	23.72 ± 7.22	23.05 ± 7.34	−1.213	0.226
Score on DASS-21 depression subscale^a^	8.82 ± 4.56	7.28 ± 4.40	−4.211	<0.001
Score on DASS-21 anxiety subscale^a^	9.79 ± 4.4	7.99 ± 5.27	−4.591	<0.001
Score on DASS-21 stress subscale^a^	8.55 ± 4.56	7.13 ± 4.67	−3.780	<0.001
Score on Insomnia Severity Index	9.02 ± 4.78	10.28 ± 5.39	3.146	0.002
Weekly hours of internet gaming	17.71 ± 5.06	17.99 ± 4.98	0.701	0.484

a Measured using Depression Anxiety Stress Scale (DASS-21).

**Table 2:** Correlation matrix among tested variables.

x	r									
	1.	2.	3.	4.	5.	6.	7.	8.	9	10
1. IGDS-SF (Adolescent)	–	0.311^**^	0.261^**^	0.180^**^	0.331^**^	0.175*	0.256^**^	0.147*	0.261^**^	0.113*
2. IGDS-SF (Sibling)		–	0.225^**^	0.370^**^	0.140*	0.372^**^	0.172^**^	0.366^**^	0.214^**^	0.338^**^
3. Depression (Adolescent)^a^			–	0.172^**^	0.452^**^	0.193^**^	0.152^**^	0.061	0.059	0.129*
4. Depression (Sibling)^a^				–	0.091	0.509^**^	0.042	0.139*	0.011	0.149^**^
5. Anxiety (Adolescent)^a^					–	0.126*	0.071	−0.062	0.124*	0.162^**^
6. Anxiety (Sibling)^a^						–	0.062	0.253^**^	−0.002	0.111*
7. Stress (Adolescent)^a^							–	0.169^**^	−0.028	0.107
8. Stress (Sibling)^a^								–	0.057	0.140*
9. ISI (Adolescent)									–	0.193^**^
10. ISI (Sibling)										–

*p < 0.05; **p < 0.01.

IGDS-SF = Internet Gaming Disorder Scale-Short Form; ISI = Insomnia Severity Index.

a Measured using the Depression Anxiety Stress Scales-21 (DASS-21).

### 3.2 APIM in depression

The results of the distinguishability test show that the model with distinguishable members differed from that with indistinguishable members (χ^2^ = 168.342, p < 0.001). Therefore, distinguishability existed between students and their siblings. Therefore, the APIM model is supported for model testing, in which model parsimony can be considered for increasing model power.

**Fig. 1:**
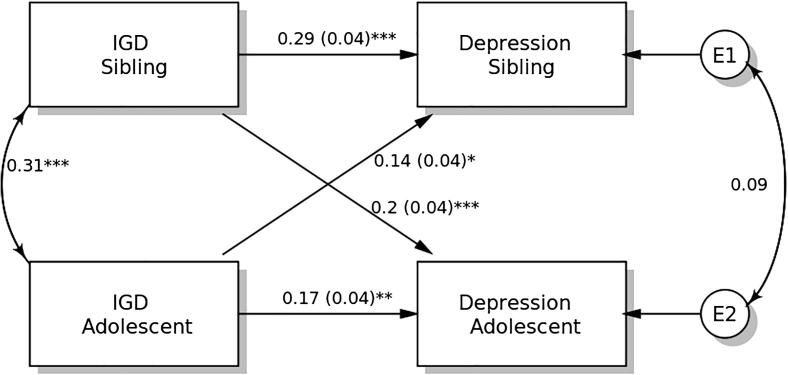
Actor-Partner Interdependence Model of the relation between internet gaming disorder (IGD) symptomatology and depression in adolescent students and their siblings. * p < 0.05; ** p < 0.01; *** p < 0.001.

IGDS-SF9 scores demonstrated significant actor effects on depression in both students and their siblings (Fig. 1). The standardized actor effect was 0.17 (SE = 0.04; p = 0.004) for adolescent students and 0.29 (SE = 0.04; p < 0.001) for their siblings, indicating that higher IGD scores in both adolescent students and their siblings predicted their own depression level. Partner effects on depression were significant in both adolescent students (standardized effect = 0.14; SE = 0.04; p = 0.012) and their siblings (standardized effect = 0.20; SE = 0.04; p < 0.001).

### 3.3 APIM in anxiety

The results of the distinguishability test show that the model with distinguishable members differed from that with indistinguishable members (χ^2^ = 157.802, p < 0.001). Therefore, distinguishability existed between students and their siblings. Therefore, the APIM model is supported for model testing, in which model parsimony can be considered for increasing model power.

**Fig. 2:**
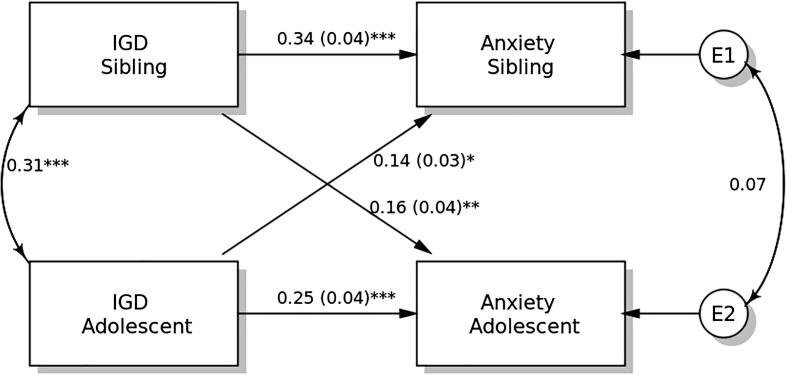
Actor-Partner Interdependence Model of the relation between internet gaming disorder (IGD) symptomatology and anxiety in adolescent students and their siblings. * p < 0.05; ** p < 0.01; *** p < 0.001.

IGDS-SF9 scores demonstrated significant actor effects on anxiety in both students and their siblings (Fig. 2). The standardized actor effect was 0.25 (SE = 0.04; p < 0.001) for students and 0.34 (SE = 0.04; p < 0.001) for their siblings, indicating that higher IGD scores in both adolescent students and their siblings predicted their own anxiety level. Partner effects on the anxiety were significant in both students (standardized effect = 0.14; SE = 0.03; p = 0.014) and their siblings (standardized effect = 0.16; SE = 0.04; p = 0.004).

### 3.4 APIM in stress

The results of the distinguishability test show that the model with distinguishable members differed from that with indistinguishable members (χ^2^ = 166.917, p < 0.001). Therefore, distinguishability existed between students and their siblings. Therefore, the APIM model is supported for model testing, in which model parsimony can be considered for increasing model power.

**Fig. 3:**
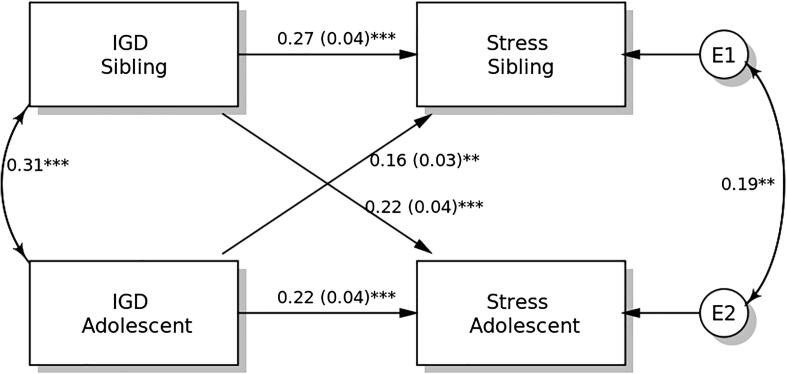
Actor-Partner Interdependence Model of the relation between internet gaming disorder (IGD) symptomatology and stress in adolescent students and their siblings. * p < 0.05; ** p < 0.01; *** p < 0.001.

IGDS-SF9 scores demonstrated significant actor effects on stress in both students and their siblings (Fig. 3). The standardized actor effect was 0.22 (SE = 0.04; p < 0.001) for students and 0.27 (SE = 0.04; p < 0.001) for their siblings, indicating that higher IGD scores in both students and their siblings predicted their own stress level. Partner effects on the stress were significant in both students (standardized effect = 0.16; SE = 0.03; p = 0.004) and their siblings (standardized effect = 0.22; SE = 0.04; p < 0.001).

### 3.5 APIM in insomnia

The results of the distinguishability test show that the model with distinguishable members differed from that with indistinguishable members (χ^2^ = 30.072, p = 0.006). Therefore, distinguishability existed between students and their siblings. Therefore, the APIM model is supported for model testing, in which model parsimony can be considered for increasing model power.

**Fig. 4:**
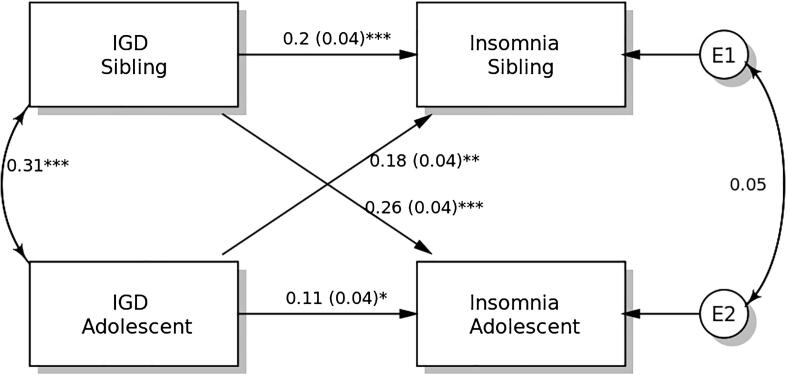
Actor-Partner Interdependence Model of the relation between internet gaming disorder (IGD) symptomatology and insomnia in adolescent students and their siblings. * p < 0.05; ** p < 0.01; *** p < 0.001.

IGDS-SF9 scores demonstrated significant actor effects on insomnia in both students and their siblings (Fig. 4). The standardized actor effect was 0.11 (SE = 0.04; p = 0.047) for adolescent students and 0.20 (SE = 0.04; p < 0.001) for their siblings, indicating that higher IGD scores in both adolescent students and their siblings predicted their own insomnia severity. Partner effects on the insomnia were significant in both students (standardized effect = 0.18; SE = 0.04; p = 0.001) and their siblings (standardized effect = 0.26; SE = 0.04; p < 0.001).

## 4 Discussion

To our best knowledge, no studies have examined interrelationships between IGD and psychological distress/insomnia among adolescent students and their siblings. With all hypotheses supported, the present study’s APIM findings suggest that impacts of IGD on psychological distress and insomnia severity exist in at least two routes. One route is within actors (i.e., within adolescent students themselves and within the siblings themselves) and corroborates prior findings that IGD may lead to psychological distress (Andreassen et al., 2016, Cole and Hooley, 2013, Hyun et al., 2015, King and Delfabbro, 2016, Laconi et al., 2017, Wang et al., 2019) and sleep problems (Alimoradi et al., 2019, Fossum et al., 2014, Wong et al., 2020). The second route is between actors and partners (i.e., between adolescent students and their siblings and between siblings and adolescent students) and routes between actors and partners has not been previously examined.

After comparing scores on the IGDS-SF9 (Mean score = 24.0; Wu et al., 2017), DASS-21 (Mean score = 7.6 in depression, 8.2 in anxiety, and 7.6 in stress; Wu et al., 2017) in the prior Iranian adolescent research, the present sample experienced similar level of severity of IGD (t = 1.51; p = 0.13), depression (t = 1.80; p = 0.07), and stress (t = 0.93; p = 0.35), but significantly more anxiety (t = 2.55; p = 0.01). When using the suggested cutoffs for psychological distress (scores between 10 and 13 indicate mild depression; between 8 and 9 indicate mild anxiety, and between 15 and 18 indicate mild stress) and insomnia (scores between 8 and 14 indicate subthreshold insomnia), the present sample experienced subthreshold/non-significant psychological distress and a mild level of insomnia.

Significant findings between actors and partners may be explained in several ways. First, per attachment theory (Ainsworth, 1989, Bowlby, 1969), siblings are a basis of security because they usually support and encourage each other (Mota & Matos, 2015), especially during adolescence, a period when adolescents individuate from their parents (Pace & Zappulla, 2013). Therefore, adolescents and their siblings may share common interests (e.g., gaming) and exchange emotions (Dunn, 2000). With gaming and emotional attachments, each others’ severities of IGD impact on each others’ psychological distress. Moreover, adolescents may feel responsible for their siblings’ IGD symptomatology and this may result in psychological distress. Specifically, if an adolescent observes his or her sibling having gaming problems, the adolescent may self-blame and develop psychological distress. However, further research is needed to investigate these possibilities.

Second, given that the recruited participants (adolescents and their siblings) share the same bedroom, their living patterns were likely to be interrelated. With interrelated living patterns, their gaming behaviors, especially gaming in the bedroom, could possibly impact each other’s sleep. For example, when an adolescent student delays bedtime because of gaming, the adolescent may have elevated mood (Wong et al., 2020) and subsequently increase his or her sibling’s mood or state of arousal. Sleepiness at bedtime may thus be reduced by psychological stimulation (Hale et al., 2018). Moreover, gaming may make the environment nonconducive for sleeping (e.g., noise and light from gaming). Therefore, with either the adolescent or his/her sibling gaming, the bedroom may not be comfortable for promoting sleep; subsequently, both the adolescent and sibling may experience poor qualities of sleep.

With the novel findings in the mutual influences on the IGD and psychological well-being between adolescents and their siblings, healthcare providers should consider this relationship when they design programs focusing on IGD and psychological well-being. Specifically, healthcare providers may wish to understand whether an adolescent enrolled into an IGD reduction or psychological well-being improvement program has a sibling. If the adolescent has a sibling, such a program may consider assessing the adolescent’s sibling and potential influences between the adolescent and his/her sibling. This approach may help optimize treatment impact.

Several study limitations exist. First, the cross-sectional design cannot provide insight into causal relationships. Associations between internet gaming and psychological distress may be reciprocal. The Interaction of Person-Affect-Cognition-Execution (I-PACE) model proposes that an individual may develop addictive behaviors (e.g., IGD) when he or she has psychological distress under a stressful environmental context (e.g., academic and peer pressures for adolescent students) (Brand et al., 2019). Empirical evidence from Gentile et al. (2011) showed that levels of IGD at baseline contributed to depression and anxiety at a 2-year follow-up. Although the relationship between IGD and psychological distress could be reciprocal, the present cross-sectional study investigated the direction from IGD to psychological distress. A reason for making this assumption is we assume that the present study’s participants have already gone through the stages described in the I-PACE. That is, they had already developed gaming habits. Therefore, we focused on the potential impacts of IGD on psychological distress. In addition, two recent studies using a longitudinal design found that internet addiction may lead to psychological distress (Chen et al., 2020a, Yu and Shek, 2018). Thus, we believe that the hypothesized models in the present study have support. Nevertheless, future studies using longitudinal designs are warranted. Second, measures used were self-reported. Hence, the data are subject to biases relating to social desirability, common method variance, and recall. Third, some potentially important factors relating to IGD, psychological distress, and insomnia were not collected and thus could not be considered in statistical analyses. For example, parenting styles and parents’ monitoring of screen use are likely to influence adolescents’ and siblings’ gaming behaviors. Fourth, participants were recruited from a single city (Qazvin) in Iran. Therefore, generalizability of findings is limited. Fifth, we did not use a structured interview for IGD diagnosis. Therefore, we cannot verify whether any of the participants had formal IGD diagnoses. Lastly, some of the instruments were designed for adults, and it is unclear whether these instruments maintain their strong psychometric properties when assessing adolescent populations.

## 5 Conclusion

To conclude, the present study used an APIM to interrogate dyadic data from adolescent students and their siblings to investigate within- and across-dyad impacts of IGD on psychological health and sleep. According to the present findings, healthcare providers, parents, teachers and other stakeholders should consider siblings when targeting IGD in adolescents. Related interventions may consider involving adolescents and their siblings.

## 6 Author statement**a

The study was conceived and designed by AHP and CYL. CYL and AHP wrote the first draft of the manuscript. MNP and AB provided critical feedback on the first draft and contributed to writing of subsequent drafts. All authors contributed significantly to and edited all sections of the manuscript and have approved the final version.

## Declaration of Competing Interest

The authors declare that they have no known competing financial interests or personal relationships that could have appeared to influence the work reported in this paper.
